# Interleukin-10 levels in azithromycin-induced cardiac damage and the protective role of combined selenium and vitamin E treatment

**DOI:** 10.1016/j.toxrep.2024.101860

**Published:** 2024-12-10

**Authors:** Heba Hussein Rohym, Mohamed S. Hemeda, Almoatazbellah Mahmoud Elsayed, Mayada Saad Farrag, Heba A. Elsayed, Amgad A. Ezzat, Mohamed A. Ibrahim, Mohammed Makloph

**Affiliations:** a Department of Forensic Medicine and Clinical Toxicology, Fayoum University, Egypt; b Department of Forensic Medicine and Clinical Toxicology Faculty of Medicine, Port Said University, Port Said, Egypt; c Department of Basic Medical and Dental Sciences, Faculty of Dentistry, Zarqaa University, Zarqaa, Jordan; d Department of Pathology, Faculty of Medicine, Port Said University, Port Said, Egypt; e Department of Microbiology, Faculty of Medicine, Port Said University, Port Said, Egypt; f Medical Microbiology and Immunology, Faculty of Medicine for Boys (Assiut), Egypt; g Department of Pathology, Faculty of Medicine, Al-Azhar University, Assiut, Egypt

**Keywords:** Azithromycin, Heart, Interleukin-10, Selenium, Vitamin E

## Abstract

Azithromycin is a broad-spectrum antibiotic commonly used to treat bacterial infections but is associated with adverse cardiac effects, including oxidative damage and myocardial inflammation. This study aims to explore the histopathological and biochemical changes, including serum interleukin-10 levels, induced by azithromycin in the hearts of male albino rats and to evaluate the protective role of combined selenium and vitamin E treatment. Forty rats were divided into four groups: a control group, an azithromycin treatment group, selenium and vitamin E treatment group, and a combined treatment group receiving both azithromycin, selenium, and vitamin E. Results showed that the azithromycin-treated group exhibited significant increases in interleukin-10 levels, myocardial fibrosis, and cell structure degeneration, while combined selenium and vitamin E treatment markedly reduced these adverse effects, indicating a protective effect. This study concludes that selenium and vitamin E provide a protective effect against azithromycin-induced cardiac toxicity, suggesting that concurrent antioxidant therapy may help safeguard the heart during azithromycin treatment.

## Highlights

• Azithromycin administration in male albino rats results in significant cardiac damage, including myocardial fibrosis, inflammation, and elevated Interleukin-10 levels.
• Selenium and Vitamin E co-administration effectively reduces Azithromycin-induced cardiotoxicity by restoring antioxidant enzyme levels and reducing oxidative stress markers.
• Histological analysis revealed that Selenium and Vitamin E mitigate myofiber disorganization and inflammatory infiltration in cardiac tissues treated with Azithromycin.
• Immunohistochemical findings showed decreased levels of caspase 3 and TNF-α in the group receiving Selenium and Vitamin E, suggesting reduced apoptosis and inflammation.
• These findings support the potential of antioxidant therapy as a protective strategy against the cardiotoxic effects of Azithromycin, highlighting the therapeutic role of Selenium and Vitamin E.

## Graphical Abstract

**Figure:**
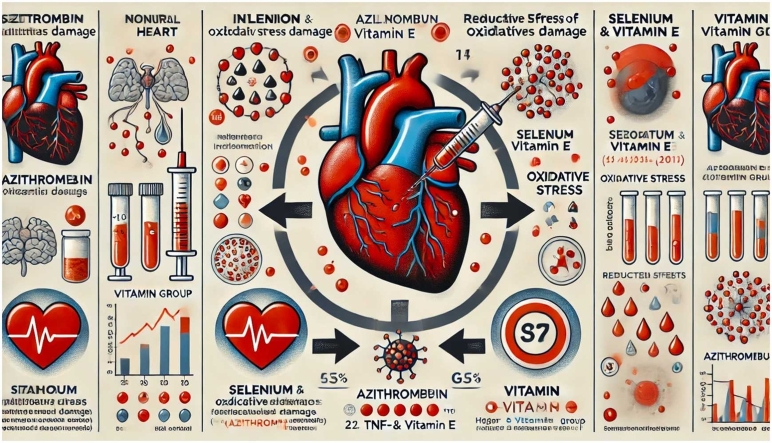

## 1 Background

Azithromycin is an antibiotic that fights bacterial infections. It is widely used to treat various bacterial diseases. These include respiratory, skin, vision, eye, and sexually transmitted infections. It has been shown that azithromycin stops viruses from getting into cells and boosts the body's defense against bacteria by increasing the production of type I and III interferons, especially interferon-β and interferon-κ, and downregulating genes that help the body recognize viruses [1].

The antiviral activity and immunomodulatory effects of these factors substantiate the drug's potential efficacy in COVID-19 infection [2].

A macrolide antibiotic called azithromycin (AZ) is often given as the first treatment for people with acquired immunodeficiency syndrome (AIDS) to stop and control the spread of Mycobacterium avium-intracellular infections [3]. However, numerous reports have highlighted the potential cardiac risks associated with azithromycin, including incidents of Torsades de Pointes and sudden cardiac death from ventricular arrhythmias in patients receiving AZ treatment. The increased risk of cardiac complications is associated with a history of heart disease, high doses, rapid administration, elevated blood concentrations, advanced age, and cardiovascular risk factors such as diabetes. Azithromycin cardiotoxicity has been shown to increase the risk of mortality due to cardiac arrhythmias and sudden death. Studies in rat models show that azithromycin can cause oxidative damage, inflammation, and cell death in heart tissue [4]. This led to the discovery of an abnormal electrocardiogram, myocardial infarction, and death [5].

Several potential mechanisms may be responsible for the azithromycin-induced cardiotoxic effects. (1) Interaction of azithromycin with cardiac calcium and potassium ion channels: The long QT syndrome is a measurement of the time that the heart takes to repolarize after each heartbeat. Prolonged QT intervals are known to increase the risk of the appearance of life-threatening arrhythmias. Azithromycin has been reported to prolong the QT interval in a few cases. (2) Production of free radicals that are responsible for lipid peroxidation and tissue or cell damage. The generation of oxidative stress is a trigger for several cardio-toxic or non-cardio-toxic compounds. (3) Generation of inflammatory signaling through myocardial inflammation. Acting as a stressor, azithromycin has been reported to increase inflammation in patients. (4) Genetic predisposition: The patient's genetic makeup may partially explain the existence of people vulnerable to drugs [6].

Vitamin E, a fat-soluble vitamin, consists of four tocopherols (α, β, δ, γ) and four tocotrienols (α, β, δ, γ). This vitamin is essential for the formation of red blood cells and preventing cell oxidation. It is abundant in whole grains, wheat germ, green leafy vegetables, scrambled eggs, and sardines. In addition to its antioxidant activity, vitamin E has shown antioxidant activity, including the ability to improve environmentally induced tissue changes in rat myocardial pollutants and drugs such as azithromycin [7]. These effects are attributed to its regulation of oxidative stress, inhibition of red cell degeneration in hemostasis, and inhibition of lipid peroxidation. It has also been shown that vitamin E can protect against bleomycin hydrochloride-induced pulmonary fibrosis and cisplatin-induced inflammatory nephrotoxicity [8].

Selenium, an important tiny part for both people and animals, is mostly seen tied to selenoproteins. These selenoproteins, like glutathione peroxidases, have a key job in shielding cells from harm caused by too many reactive oxygen types (ROS) [9]. A low selenium amount in people has been linked with a higher chance of many sicknesses, including heart problems. The mix of selenium and vitamin E has been suggested as a possible way to fight oxidative hurt, especially in heart tissue [5].

## 2 Methods

### 2.1 Chemicals

1. Azithromycin and vitamin E were acquired from Pharco Co. in Alexandria, Egypt.
2. Selenium was purchased from Sigma Chemical Co. It was provided in the form of a white powder and diluted with 0.9 % sodium chloride [9].

#### 2.1.1 Compliance with ARRIVE guidelines

All animal protocols should be approved and conducted by the Institutional Animal Care and Use Committee.

By the ARRIVE (Animal Research: Reporting of In Vivo Experiments) guidelines, all animal handling and experimental procedures were conducted with the highest standards to ensure ethical treatment and scientific rigor.

1. **Animal Housing and Welfare**: The animals were housed individually in standard laboratory conditions, with a controlled temperature of 22 ± 2°C, a 12-hour light/dark cycle, and unrestricted access to food and water. Animal welfare was monitored daily by trained personnel to ensure health and well-being, and humane endpoints were established to minimize distress.
2. **Randomization and Blinding**: Animals were randomly distributed to each experimental group using a random number generator to guarantee unbiased group allocation. Additionally, individuals involved in data collection and analysis were not aware of the groups. Treatment to avoid potential bias in interpreting results
3. **Sample Size and Statistical Power**: The sample size (n = 10) for each group was determined based on power calculations. This ensured that statistically significant differences could be detected between groups. This resulted in a reduction in the number of animals used while maintaining adequate statistical precision.
4. **Outcome measures and statistical analysis:** Primary and secondary outcome measures are clearly defined. Including histopathological evaluation. Biochemical markers and antioxidant levels Statistical analysis was performed using ANOVA followed by Tukey's HSD test to assess differences between groups. Statistical significance was set at P < 0.05.

### 2.2 Experimental design and animals

Forty adult male albino rats (Sprague) weighing between 180 and 220 g were used in this study. The animals were obtained from the animal research facility of the Assiut University Animal Research Center according to ethical guidelines for animal welfare and research. The facility confirmed that it keeps animals in wire cages that are well-ventilated and at ambient temperature, with unrestricted access to food and water. Two weeks of acclimatization were allowed before the trials. The rats were then randomly divided into four groups, each with 10 rats weighing between 180 and 220 g. The study was performed at the animal house of the Pharmacology Department, Faculty of Medicine, Fayoum University.

Forty adult male albino rats were divided into four groups (10 rats per group) as follows:

• **Group I (Control)**: Received distilled water intragastrically without any treatment.
• **Group II (Azithromycin only)**: Rats were administered azithromycin intragastrically at a dose of 30 mg/kg/day for two weeks, with no additional supplements.
• **Group III (Selenium and Vitamin E only)**: Rats received selenium (10 µg/kg/day) and vitamin E (20 mg/kg/day) intragastrically for two weeks, without azithromycin.
• **Group IV (Combination of Azithromycin, Selenium, and Vitamin E)**: Rats were administered azithromycin (30 mg/kg/day), along with concurrent administration of selenium (10 µg/kg/day) and vitamin E (20 mg/kg/day) for two weeks.

Twenty-four hours after the final dose, rats were anesthetized by intraperitoneal injection of ketamine (60 mg/kg) and xylazine (5 mg/kg). The hearts were excised, and the animals were sacrificed via cervical dislocation. The hearts were then prepared for subsequent histopathological, biochemical, and immunohistochemical investigations.

### 2.3 Chemicals and materials

The following chemicals and materials were used in this study, with sources specified to ensure reproducibility and transparency 10,11,12,13:

1. **Azithromycin**: Sourced from Pharco Co., Alexandria, Egypt.

**Preparation**: Azithromycin, acquired as a powdered form from Pharco Co., Alexandria, Egypt, was prepared by dissolving 300 mg of the powder in 15 ml of distilled water to create a 20 mg/ml solution.

**Dosage and Administration**: The azithromycin solution was administered intragastrically at a dose of 30 mg/kg/day. Each animal's weight was measured, and the appropriate volume of solution was calculated based on body weight to ensure accurate dosing.

**Dosage Spacing**: Azithromycin was administered to the groups sequentially once daily for a consecutive period of two weeks. Schedule a regular time every day to maintain a consistent level of healing.

2. **Vitamin E**: obtained from Pharco Company of Alexandria, Egypt. Vitamin E was administered at a dose of 20 mg/kg/day.

**Preparation**: Use vitamin E obtained from Pharco dissolved in oil. Prepared according to the manufacturer's instructions to ensure an appropriate concentration for gastrointestinal administration.

**Dosage and Administration**: Vitamin E is given at a dose of 20 mg/kg/day. The weight of each animal is used to calculate the exact dosage.

**Dosage interval**: Give once-daily dosing. By providing selenium For two weeks with the group involved.

3. **Selenium is purchased from Sigma Chemical Company.**

**Preparation**: Selenium was purchased from Sigma Chemical as a white powder. The powder was diluted in 0.9% sodium chloride solution to obtain the concentration required for quantification.

**Dose and Administration**: Selenium is administered at a dose of 10 mcg/kg/day. The dose volume for each mouse was calculated separately based on body weight to ensure accuracy.

**Dosage interval**: Selenium is given daily for two weeks by injection into a single dose per day.

4. **Anesthesia - Ketamine and Xylazine**: Ketamine (60 mg/kg) and xylazine (5 mg/kg) are used as anesthesia. It is given intraperitoneally to ensure humane treatment and proper anesthesia before the animal is sacrificed.

5. **Histological and Immunohistochemical Staining Reagents:**

• **Hematoxylin and eosin (H&E)**: Used for general cardiac tissue staining of cardiac tissue sections to monitor structural changes.

Caspase-3 and TNF-α antibodies: These antibodies were used in immunohistochemical staining to assess cell death and inflammatory markers in heart tissue and the slides were analyzed using software. FIJI image processing

6. **Biochemical Assay Kits**:

• Creatine Kinase (CK-MB) and Lactate Dehydrogenase (LDH) kit: used for biochemical evaluation of cardiac injury markers according to manufacturer's instructions.
• Total Antioxidant Capacity (TAC), Glutathione (GSH), Superoxide Dismutase (SOD), Catalase (CAT) and Malondialdehyde Assays (MDA): These tests are used to measure oxidative stress and antioxidant levels in heart tissue.
• Interleukin-10 (IL-10) ELISA Kit: This kit is used to quantify serum IL-10 levels in blood samples. Blood samples were taken from the right ventricle of anesthetized animals and allowed to rest at room temperature for 30 min. The samples were then centrifuged at 2000 × g for 15 min at 4°C. Creatin levels were measured. CKinase (CK)-MB and lactate dehydrogenase (LDH) according to the manufacturer's instructions. Heart tissue samples were mixed to determine levels of antioxidant enzymes such as glutathione (GSH), superoxide dismutase (SOD), catalase (CAT), and malondialdehyde. (MDA) and others in a ratio of 20 (w/v). The homogenate was then centrifuged at 10,000 × g for 15 min at +4°C. The supernatant was used for biochemical analysis of the levels. GSH, CAT, SOD and MDA According to the established regulations. All chemicals and reagents were controlled according to the manufacturer's protocol to maintain consistency throughout the entire experimental procedure.

**Dosing Method and Precautions:** All compounds are administered by injection into the gastrointestinal tract to ensure complete absorption and control of the administered dose. Intraperitoneal anaesthesia with ketamine and xylazine was used as needed before terminal procedures to minimize animal distress. Doses were prepared fresh daily, and administration was done at the same time each day to maintain consistency. Animals were monitored continuously to check for any adverse reactions post-dosing, and doses were adjusted based on the precise weight of each animal at the start of the treatment period.

### 2.4 Statistical analysis

All data were double-entered in Microsoft Access and analyzed using SPSS 22 (Statistical Package for the Social Sciences) for Windows 7. Results were expressed as mean ± standard deviation (SD). For comparisons between the different experimental groups, a one-way analysis of variance (ANOVA) was used to assess the significance of differences across groups. We used Tukey's Honestly Significant Difference (HSD) test to find statistical significance between all relevant pairs in the 2 × 2 experimental design after the fact to compare specific groups pairwise. The following comparisons were made:

• Group I (Control) vs. Group II (Azithromycin Treatment)
• Group I (Control) vs. Group III (Selenium and Vitamin E Treatment)
• Group I (Control) vs. Group IV (Azithromycin with Selenium and Vitamin E Treatment)
• Group II (Azithromycin Treatment) vs. Group III (Selenium and Vitamin E Treatment)
• Group II (Azithromycin Treatment) vs. Group IV (Azithromycin with Selenium and Vitamin E Treatment)
• Group III (Selenium and Vitamin E Treatment) vs. Group IV (Azithromycin with Selenium and Vitamin E Treatment)

Statistical significance was set at *P* < 0.05 for all comparisons. Results with a *P* < 0.05 were considered statistically significant and were indicated in the tables and figures using an asterisk (*).

### 2.5 Ethical considerations

All experimental procedures were approved by the Faculty of Medicine's Ethics Committee at Fayoum University (R 309) and were conducted in a controlled laboratory setting at the Pharmacology Department animal house at the Fayoum University, Faculty of Medicine.

## 3 Results

### 3.1 Histopathological findings

**Fig. 1:**
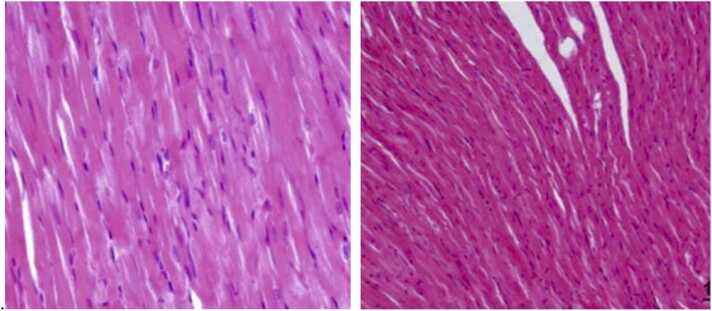
Groups I (Control) and III (Selenium and Vitamin E Treatment): The examination of heart tissues under a light microscope reveals normal morphological appearances, devoid of any visible signs of myofiber degeneration or inflammatory infiltration. We used Hematoxylin and Eosin (H&E) staining at a 400x magnification.

**Fig. 2:**
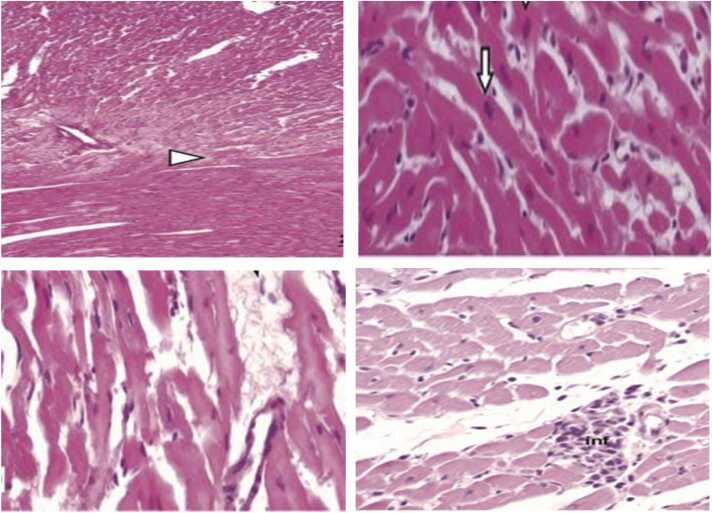
Group II (Azithromycin Treatment): A light microscope looks at heart ventricle tissue reveals a lot of myofiber disorganization, with gaps between fibers that can be seen, as well as areas of inflammation and bleeding. These findings indicate marked degeneration due to azithromycin administration. We used Hematoxylin and Eosin (H&E) staining at a 400x magnification.

Light microscopic examination of heart tissues in control groups (Group I, Group III, and Group IV) revealed normal morphological appearances (Fig. 1). In contrast, Group II, treated with azithromycin, showed significant degeneration in the heart's ventricular tissues. This included disorganized myofibers with large gaps, inflammatory infiltration, and areas of hemorrhage, as shown in Fig. 2.

**Fig. 3:**
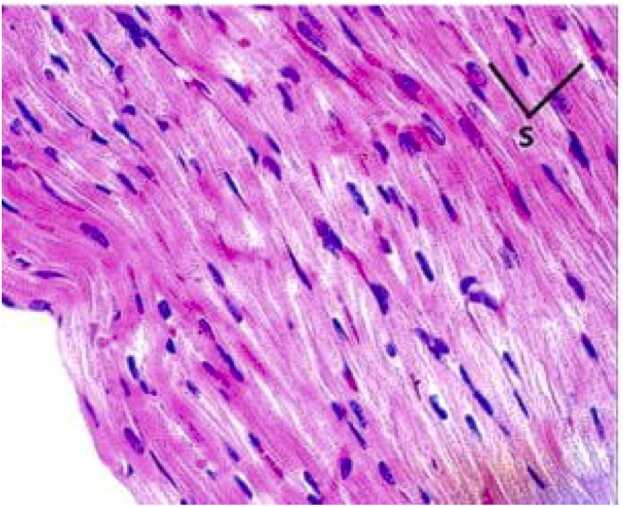
Group IV (Azithromycin with Selenium and Vitamin E Treatment): A close look through a light microscope at the heart tissues shows that they have returned to their normal shape, just like the control group. This suggests that selenium and vitamin E are protecting the heart from damage caused by azithromycin. We used Hematoxylin and Eosin (H&E) staining at a 400x magnification.

The histological examination of cardiac tissues in Group IV (azithromycin with selenium and vitamin E treatment) is shown in Fig. 3. The combined administration of selenium and vitamin E with azithromycin resulted in a protective effect on the heart tissue, as evidenced by the preservation of normal myocardial structure and the reduction of inflammatory infiltration. In contrast, Group II (azithromycin-only) exhibited significant myocardial degeneration, including disorganization of muscle fibers, cellular inflammation, and cytoplasmic vacuolization. The marked restoration of normal tissue morphology in Group IV highlights the potential of selenium and vitamin E to mitigate azithromycin-induced cardiac toxicity.

### 3.2 Immunohistochemical evaluation

**Fig. 4:**
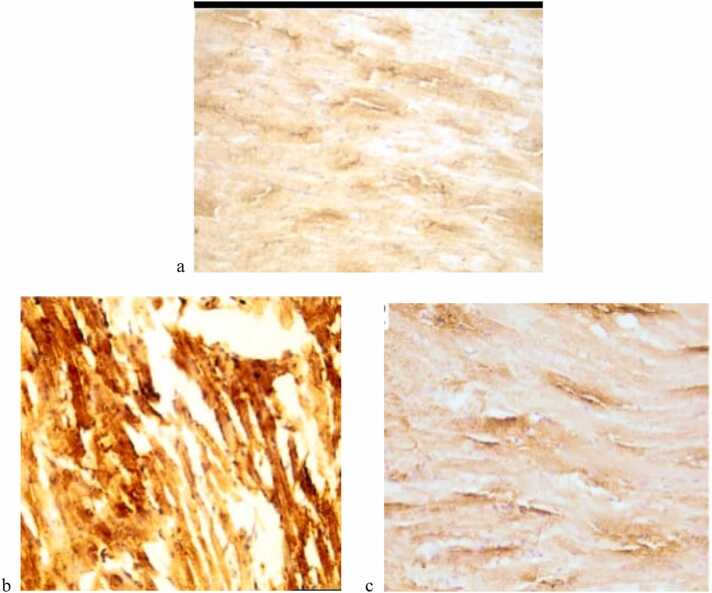
(a, b, c) shows the immunohistochemical staining for Caspase-3 in heart ventricle tissue. 4a: There aren't many positive cytoplasmic immune reactions in Group I (Control) or Group III (Selenium and Vitamin E Treatment), which means there aren't many cell apoptosis events. 4b: Group II (Azithromycin Treatment) shows a strong positive cytoplasmic immune reaction, highlighting increased apoptosis due to azithromycin exposure. 4c: Group IV (Azithromycin with Selenium and Vitamin E Treatment) has few positive immune reactions, which suggests that giving these drugs together lowers the death of cells. Staining is observed at 400x magnification.

**Fig. 5:**
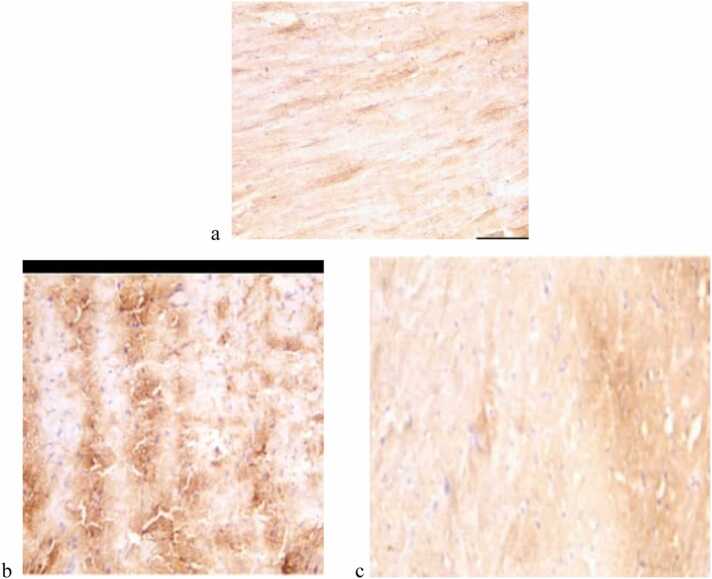
(a, b, c) shows the immunohistochemical staining for TNF- in heart ventricle tissue. 5a: There aren't many positive cytoplasmic immune reactions in Group I (Control) or Group III (Selenium and Vitamin E Treatment), which means there isn't much inflammation. 5b: Group II (Azithromycin Treatment) shows strong positive immune reactions, reflecting elevated inflammation associated with azithromycin exposure. 5c: People in Group IV (Azithromycin with Selenium and Vitamin E Treatment) have fewer positive immune responses, which suggests that the selenium and vitamin E are working together to reduce inflammation. Observations were made at 400x magnification.

The group that was given azithromycin (Group II) had significantly higher levels of both caspase-3 and tumor necrosis factor-alpha (TNF-α) immune stains compared to the control group (Group I). Co-administration of selenium and vitamin E in Group IV restored these markers to near-normal levels, as seen in Fig. 4, Fig. 5.

### 3.3 Biochemical testing

There was a big rise in CK-MB and LDH levels in the azithromycin-treated group (Group II) compared to the control group (Group I) and the other treatment groups (Group III and Group IV) (Table 1). The elevation in CK-MB and LDH levels indicates azithromycin-induced cardiac damage.

**Table 1:** CK-MB and LDH Levels (Mean ± SD) Across Study Groups.

**Group**	**CK-MB (ng/ml)**	**LDH (IU/L)**
Group I (Control)	46.8 ± 8.41	37.41 ± 11.99
Group II	67.05 ± 9.6*	60.95 ± 9.66*
Group III	46.9 ± 8.41	36.41 ± 11.43
Group IV	47.05 ± 8.41	37.11 ± 10.99

* Significant increase compared to the control group (P < 0.05).

Table 2 presents the levels of antioxidant enzymes (SOD, CAT, GSH) and MDA across the groups. Group II exhibited significantly decreased levels of GSH, SOD, and CAT, and an increase in MDA, indicating oxidative stress. Selenium and vitamin E treatment in Groups III and IV helped restore these levels.

**Table 2:** Antioxidant Enzyme Levels and MDA in Heart Tissues Across Study Groups.

**Group**	**MDA (pmol/ml)**	**GSH (µg/g Tissue)**	**SOD (U/ml)**	**CAT (nmol/min/ml)**
Group I (Control)	1080.10 ± 120.25	21.3 ± 1.07	1.53 ± 0.12	378.27 ± 24.18
Group II	1350.16 ± 135.58*	15.06 ± 1.66*	1.48 ± 0.1*	349.21 ± 18.2
Group III	1084.10 ± 118.25	20.8 ± 1.07	1.52 ± 0.22	380.27 ± 23.18
Group IV	1081.10 ± 120.25	21.1 ± 1.07	1.53 ± 0.12	377.27 ± 27.18

* Significant difference compared to control group (P < 0.05).

Table 3: Interleukin-10 serum levels (mean ± SD) measured on days 1, 7, and 14 across the four study groups. Group II (azithromycin-treated group) displayed significantly elevated IL-10 levels compared to the control group (Group I) on all measured days (*P* < 0.05). The co-administration of selenium and vitamin E in Group IV resulted in levels comparable to the control group.

**Table 3:** Interleukin-10 Serum Levels (Mean ± SD) Across Study Groups.

**Group**	**Day 1 (pg/ml)**	**Day 7 (pg/ml)**	**Day 14 (pg/ml)**
Group I (Control)	8.1 ± 0.5	8.3 ± 0.8	8.2 ± 0.5
Group II	9.6 ± 0.8*	10.13 ± 0.4*	10.03 ± 0.3*
Group III	8.0 ± 0.5	7.9 ± 1.1	7.8 ± 0.9
Group IV	8.2 ± 0.6	8.4 ± 0.7	8.6 ± 0.4

* Significant difference compared to the control group (P < 0.05).

## 4 Discussion

Numerous studies have investigated the potential cardiac side effects of azithromycin, a drug widely used to treat bacterial infections. ¹ ² Under a light microscope, hematoxylin and eosin-stained heart tissues from Group I (the control group), Group III (which got selenium and vitamin E treatment), and Group IV (which got azithromycin followed by selenium and vitamin E treatment). All of these groups looked normal. On the other hand, the azithromycin group (Group II) exhibited significant damage in the heart ventricle, including myofiber disorganization with large gaps, inflammation, and bleeding. These results are similar to what Shaimaa Mostafa Kashef and Noha Elswaidy found in 2021. They said that azithromycin caused cardiotoxicity in rats, which was shown by myocyte disintegration, lipid infiltration, and mononuclear cellular infiltration [7]. A study by Mansoor and colleagues (2021) also found that azithromycin causes extensive damage to the heart, such as degeneration, hemorrhage, and myofiber disorganization [5]. From immunohistochemical examination The azithromycin-treated group (Group 2) had caspase-3 immunoreactivity. and tumor necrosis factor-alpha (TNF-α) were much higher than those in the control group (Group 1). Caspase-3 is a well-known marker of cell death. This indicates that oxidative stress induced by azithromycin induces apoptosis in cardiomyocytes. The results of this study are consistent with those of Mansour and colleagues (2021), who found that azithromycin increases the production of reactive oxygen species (ROS), which causes cell death by activating caspase-3.4. in our study, Simultaneous consumption of selenium and vitamin E (group IV) reduced caspase-3 staining. significantly This suggests that the antibiotic reduces the effect of azithromycin on cell death. This study also showed that selenium and vitamin E can help protect against cardiac damage caused by azithromycin by lowering reactive oxygen species (ROS). This is similar to what Ahmed S's study found. Since ROS has detrimental effects on various body organs, reducing it will safeguard them [10].

TNF-α indicates cytokine involved in inflammation, and elevated levels of it in the heart muscle indicate an inflammatory response to azithromycin. The group treated with azithromycin (group II) showed severe staining for TNF-α, indicating the presence of overt inflammation. Mansour et al. (2021) similarly reported increased TNF-α levels in rats treated with azithromycin, which linked this inflammatory response to the drug's cardiac toxicity [4]. Our findings indicate that selenium and vitamin E (group IV) together contribute to normal TNF levels. This suggests that if these antibodies exert anti-inflammatory effects against the role of azithromycin-induced cardiomyopathy.

The immunomodulatory effect of azithromycin depends on the site of inflammatory activity. In the early stages of infection, azithromycin stimulates leukocyte function and enhances the immune response, while in the later stages, it suppresses inflammation and contributes to its remission [11]. However, in this study, the azithromycin-treated group (group II) showed a significant increase in malondialdehyde (MDA) levels. The study revealed a significant decrease in the levels of anti-inflammatory enzymes such as glutathione (GSH), catalase (CAT), and superoxide dismutase (SOD), a marker of lipid peroxidation and oxidative stress. This decrease in antioxidant enzymes suggests that azithromycin stimulates oxidative stress, as previously described by Wortmann et al. (2013), which has been shown to reduce antioxidant defenses in cardiac muscle cells and increase oxidative damage [8].

Lipid peroxidation, shown by higher MDA levels, is a reliable sign of oxidative stress. Its rise in group II which was treated with azithromycin shows that cardiac muscle cells are severely damaged by oxidative stress [8]. In contrast, selenium and vitamin E supplementation (groups III and IV) significantly restored antioxidant enzyme levels (GSH, CAT, and SOD) and lowered MDA levels, indicating oxidative pressure protection. These findings are consistent with previous studies that have shown selenium and vitamin E to reduce oxidative damage in the heart muscle by enhancing anti-inflammatory protection 8, 9.

Also, this was the present study showed similar results on MDA, glutathione, catalase, and superoxide dismutase, in agreement with those found by Ahmed S in his study on 50 albino rats. 15 The current study revealed elevated levels of glutathione, catalase (CAT), and superoxide dismutase (SOD) in the biochemical blood assessment, indicating the beneficial effects of selenium and vitamin E on azithromycin-induced damage. These findings were confirmed by Praseena P in his study on 50 albino rats' livers [12].

In addition, blood levels of interleukin-10 (IL-10) increased significantly in the azithromycin-treated group (group II) compared to the control group (group I) at 1, 7, and 14 days. This finding is consistent with that of Yang et al. (2020), which reported that IL-10, the anti-inflammatory cytokine, elevates at the onset of myocardial infarction as a compensatory response to inflammation [10]. Elevated levels of IL-10 in our study likely reflect a similar compensatory mechanism demonstrated in response to inflammation caused by azithromycin. However, the simultaneous intake of selenium and vitamin E (group IV) led to the normalization of IL-10 levels, further emphasizing the anti-inflammatory and cardioprotective effects.

### 4.1 Study limitations

While this study provides valuable insights into the protective effects of selenium and vitamin E against azithromycin-induced cardiac damage, several limitations should be acknowledged:

1. **Lack of Individual Selenium and Vitamin E Groups**: This study focused on the combined administration of selenium and vitamin E, without assessing the individual effects of each antioxidant separately. Although prior research supports the synergistic effect of both agents, future studies could benefit from evaluating the specific impact of each antioxidant to better understand their individual roles in cardioprotection.
2. **Animal Model Limitations**: The study utilized an albino rat model, which, while beneficial for examining biochemical and histological changes, may not fully represent the complexities of human physiology and cardiac responses. Further research involving human clinical trials or alternative animal models may be required to confirm the applicability of these findings to clinical settings.
3. **Short-Term Observation Period**: The effects observed in this study are limited to a two-week treatment and evaluation period. Long-term studies are needed to assess whether the protective effects of selenium and vitamin E persist over extended periods and to examine any potential side effects of prolonged administration.
4. **Limited Range of Biochemical Markers**: Although this study included several key markers of oxidative stress, inflammation, and cardiac damage, additional biomarkers could provide a more comprehensive understanding of the mechanisms involved. Future studies could expand the range of biochemical and molecular markers to better elucidate the protective pathways activated by selenium and vitamin E.

## 5 Conclusion

This study evaluated the effects of azithromycin on cardiac tissue in rats. and examine the possible protective effects of selenium and vitamin E. Four groups were evaluated: a control group, an azithromycin only group (Group II), a selenium and vitamin E only group (Group III), and a group f that received plus azithromycin, selenium, and vitamin E (Group IV). Results The study found that Azithromycin causes significant heart damage. with increased oxidative stress and inflammation. However, co-administration of selenium and vitamin E with azithromycin (Group 4) showed a clear protective effect. By restoring normal heart muscle structure. and reduces inflammation and oxidative damage. These findings suggest that selenium and vitamin E may have therapeutic potential to reduce azithromycin-induced cardiotoxicity.

Further clinical studies are needed to explore the potential therapeutic value of these groups in preventing azithromycin-induced cardiotoxicity in human patients.

## Financial support and sponsorship

None.

## Ethical approval

All animal handling and experimental procedures were approved by the Faculty of Medicine's Ethics Committee at Fayoum University (Approval No. R 309) and adhered to the ARRIVE guidelines for in vivo experiments to ensure ethical standards.

## Contributions

The research study was conducted by Heba Hussein Rohym, Mohamed S. Hemeda, Almoatazbellah Mahmoud Elsayed, Mayada Saad Farrag, Heba A. Elsayed, Amgad A. Ezzat, Mohamed A. Ibrahim, and Mohammed Makloph. The experimental design, data collection, analysis, and manuscript preparation were collaboratively managed. Each author contributed to the study's conception, methodology, and interpretation of findings. Mohamed S. Hemeda and Heba Hussein Rohym led the overall coordination of the project and manuscript review.

## Funding

This research received no external funding.

## CRediT authorship contribution statement

**Mayada Saad Farrag:** Data curation, Formal analysis, Funding acquisition, Investigation, Software, Supervision, Validation, Visualization, Writing – original draft, Writing – review & editing. **Heba A. Elsayed:** Conceptualization, Data curation, Formal analysis, Investigation, Methodology, Project administration, Resources, Software, Supervision, Validation, Visualization, Writing – original draft, Writing – review & editing. **Mohamed Hemeda:** Writing – review & editing, Writing – original draft, Visualization, Validation, Supervision, Software, Resources, Project administration, Methodology, Funding acquisition, Formal analysis, Data curation. **Mohammed Makloph:** Data curation, Formal analysis, Funding acquisition, Investigation, Methodology, Project administration, Resources, Software, Supervision, Validation, Visualization, Writing – original draft, Writing – review & editing. **Almoatazbellah Mahmoud Elsayed:** Conceptualization, Data curation, Formal analysis, Funding acquisition, Investigation, Supervision, Validation, Visualization, Writing – original draft, Writing – review & editing. **Mohamed A. Ibrahim:** Conceptualization, Data curation, Validation, Visualization, Writing – original draft, Writing – review & editing. **Heba Hussein Rohym:** Conceptualization, Data curation, Formal analysis, Funding acquisition, Investigation, Project administration, Resources, Software, Supervision, Validation, Visualization, Writing – original draft, Writing – review & editing. **Amgad A. Ezzat:** Conceptualization, Data curation, Formal analysis, Funding acquisition, Visualization, Writing – original draft, Writing – review & editing.

## Declaration of Competing Interest

The authors declare that they have no known competing financial interests or personal relationships that could have appeared to influence the work reported in this paper.

## Data Availability

No data was used for the research described in the article.
